# Antinociceptive Effect of a Sacro-Coccygeal Epidural of Morphine and Lidocaine in Cats Undergoing Ovariohysterectomy

**DOI:** 10.3390/vetsci9110623

**Published:** 2022-11-08

**Authors:** Amândio Dourado, Anabela Gomes, Paulo Teixeira, Luís Lobo, Jorge T. Azevedo, Isabel R. Dias, Rui Pinelas

**Affiliations:** 1 Veterinary Hospital of Porto, 4250-475 Porto, Portugal; 2 Department of Veterinary Sciences, School of Agricultural and Veterinary Sciences (ECAV), University of Trás-os-Montes e Alto Douro (UTAD), 5000-801 Vila Real, Portugal; 3 CECA—Center for Animal Science Studies, University of Porto, 4485-661 Vila do Conde, Portugal; 4 Faculty of Veterinary Medicine, Lusophone University of Humanities and Technology, 1749-024 Lisbon, Portugal; 5 Department of Animal Science, School of Agricultural and Veterinary Sciences (ECAV), University of Trás-os-Montes e Alto Douro (UTAD), 5000-801 Vila Real, Portugal; 6 CECAV—Center for Animal Sciences and Veterinary Studies, University of Trás-os-Montes e Alto Douro (UTAD), 5000-801 Vila Real, Portugal; 7 AL4AnimalS—Associate Laboratory for Animal and Veterinary Sciences, 5000-801 Vila Real, Portugal; 8 North Downs Specialist Referrals, Bletchingley RH1 4QP, UK

**Keywords:** antinociceptive, pain, epidural, locoregional anaesthesia, analgesia, cat, ovariohysterectomy

## Abstract

**Simple Summary:**

Few studies are reporting the use of lumbosacral epidural administration of lidocaine in cats for ovariohysterectomy (OHE). The 2020 global pain council guidelines of the World Small Animal Veterinary Association (WSAVA) also recommended an epidural with lidocaine for ovariectomy as a substitute analgesic method. However, unlike dogs, the spinal cord in cats extends more caudally (first sacral vertebra) and the dural sac terminates at the level of the third sacral vertebra. To reduce the risk of dural sac perforation and intrathecal injections, the sacrococcygeal (SCC) space has been proposed by some authors as a reasonable alternative approach to epidural space, since it prevents the existence of adverse effects related to the volume of solution administered in case of inadvertent intrathecal injection, considering the recommended volumes for the latter are significantly lower compared with those for epidural administration. Intraoperative heart rate, systolic blood pressure, and postoperative UNESP-Botucatu multidimensional composite pain scale (MCPS) and mechanical nociception thresholds (MNT) were used to access the antinociceptive effect of epidural sacrococcygeal administration of lidocaine with morphine in cats submitted to OHE. This study indicates that the implementation of SCC-E could be a simple and effective technique to control perioperative pain in cats submitted to OHE.

**Abstract:**

Background: A commonly described analgesic protocol for ovariohysterectomy (OHE) combines systemic opioids, sedatives, and non-steroidal anti-inflammatory drugs. However, systemic analgesia does not fully prevent perioperative visceral and somatic pain triggered by the surgical stimulus. Objectives: To compare the analgesic effects and quality of recovery of systemic analgesia with those of a sacrococcygeal epidural injection of lidocaine and morphine in cats undergoing elective OHE. Methods: Twenty domestic female cats were premedicated with dexmedetomidine (0.01 mg kg^−1^ IM) and alfaxalone (1.5 mg kg^−1^ IM) and randomly assigned to one of two analgesic protocols: methadone (0.2 mg kg^−1^ IM) in the control group CTR (*n* = 10) and methadone (0.1 mg kg^−1^ IM) + epidural lidocaine 2% (0.3 mL kg^−1^) + morphine 1% (0.1 mg kg^−1^) diluted with NaCl 0.9% to a total volume of 1.5 mL in the SCC-E group (*n* = 10). General anaesthesia was induced with alfaxalone (1 mg kg^−1^ IV) and maintained with sevoflurane in 100% oxygen. Non-invasive blood arterial pressure and cardiorespiratory variables were recorded. The quality of recovery was assessed using a simple descriptive scale. Before surgery and 1, 2, 3, 4, 6, and 8 h post-op pain was assessed using the UNESP-Botucatu multidimensional composite pain scale (MCPS) and mechanical nociception thresholds (MNT). The repeated measures analysis of variance (ANOVA) was used to compare groups over time. Comparison between groups was performed using independent samples *t*-test if the assumption of normality was verified, or the Mann–Whitney test. The chi-square test of independence and exact Fisher’s test were used to compare groups according to recovery quality. Results: Heart rate and systolic arterial pressure increased significantly from baseline values in the CTR group and did not change in the SCC-E group. In the CTR group, MNT and UNESP-Botucatu-MCPS scores increased significantly from baseline for all assessment points and the first 3 h, respectively, whereas this did not occur in the SCC-E group. Conclusions and clinical relevance: Based on our results, the SCC-E administration of lidocaine 2% with morphine 1% is a reasonable option to provide perioperative analgesia in cats submitted to OHE, compared to a systemic protocol alone.

## 1. Introduction

Ovariohysterectomy (OHE) is one of the most common elective surgical procedures performed in the veterinary clinical practice of companion animals [1].

Open midline approach OHE is presumed to trigger two types of pain, each one with different origins: visceral peritoneal and somatosensory pain [2,3,4,5]. Visceral peritoneal pain is the main source of nociceptive stimulation during the intraoperative period and reaches its peak during traction and ligation of the ovarian pedicles and uterus, thought to be the maximum surgical stimulus [6,7,8]. Somatosensory pain, on the other hand, is caused by trauma to the abdominal wall by the celiotomy and surgical wound and is considered the most likely source of postoperative pain.

A commonly described analgesic protocol for this procedure combines systemic opioids, sedatives, and non-steroidal anti-inflammatory drugs [9,10,11,12]. However, systemic analgesia does not fully prevent intraoperative nociception triggered by the sympathetic nervous system [7,13,14,15,16]. Indeed, systemic analgesia with opioids modulates the synaptic neurotransmission of the nociceptive stimulus as soon as it reaches the central nervous system (CNS) but does not always prevent its transmission [17]. Additionally, the well-known adverse effects of opioids in cats such as depression, vomiting, bradycardia, dysphoria, and mydriasis [18,19] not only support the need to adopt a low opioid and patient-specific dosing regimen [19] but have also prompted other authors to propose the use of locoregional anaesthesia as a complement to or substitute for systemic analgesia in veterinary medicine.

Epidural anesthesia with local anesthetics is a reasonable alternative to systemic analgesia and is regarded by some as the gold standard to promote preventive perioperative analgesia [17], resulting in better control of perioperative pain for many surgical procedures. It blocks the transmission of nociceptive stimulus by the afferent neuronal pathways before these reach the CNS. This method is thought to be simple, affordable, and has a low incidence of complications [20,21,22].

Lidocaine is a local anaesthetic frequently used for epidural injection in short surgical procedures and promotes reversible nerve conduction blockade [20,21,22,23].

Morphine is an opiate commonly used epidurally in association with local anaesthetics and it produces analgesia of up to 24 h in cats [21,24,25,26,27].

In small animal veterinary care, the lumbosacral technique is most frequently employed to access the epidural space [20,22,28,29]. Few studies have examined the utility of lidocaine administered lumbosacrally in cats for OHE [21,23,25,26,27]. However, unlike dogs, the spinal cord in cats extends more caudally (first sacral vertebra) and the dural sac terminates at the level of the third sacral vertebra [30,31]. To reduce the risk of dural sac perforation and intrathecal injections, the sacrococcygeal (SCC) space has been proposed by some authors as a reasonable alternative approach to epidural space, Refs. [32,33,34,35,36] since it prevents the existence of adverse effects related to the volume of solution administered in case of inadvertent intrathecal injection, considering the recommended volumes for the latter are significantly lower compared with those for epidural administration.

To our knowledge, this study is the first to report the epidural sacrococcygeal administration of lidocaine with morphine in cats for analgesia during OHE.

This study aimed to compare the analgesic effects and quality of postoperative recovery after a combination of methadone and dexmedetomidine with those of a combination of methadone, dexmedetomidine, and a sacrococcygeal epidural injection of lidocaine and morphine in cats undergoing elective OHE.

The authors hypothesised that compared to a systemic analgesic protocol alone, epidural SCC promotes better control of intraoperative nociception, provides superior postoperative analgesia, less perioperative opioid requirement, and better quality of anaesthetic recovery with minimal side effects.

## 2. Material and Methods

### 2.1. Animals

This animal study protocol was conducted according to national legislation on the protection of animals used for scientific purposes and with the approval of the Institutional Animal Care and Ethics Committee of the University of Trás os Montes e Alto Douro (UTAD) (protocol no. 381-e-HV-2018). After written informed consent was obtained from the cat owners, twenty domestic shorthair adult queens scheduled for elective OHE through midline laparotomy using a modified three-clamp technique were enrolled in this prospective, randomised, and blinded clinical study.

A comprehensive physical examination and a blood analysis were part of the preoperative screening.

Cats classified according to the American Society of Anaesthesia (ASA) as physical status ≥III, weighing <2 kg or >4 kg, aged <3 months or >5 years, with a body condition of <5 or >8 (1–9), having been administered any medications in the previous 72 h, pregnant or lactating were excluded. Temperamental cats that resisted a complete clinical examination or blood collection were also excluded.

The cats were admitted to the Veterinary Hospital of Porto the day before surgery to acclimatize to the environment and staff, additionally being housed in individual cages in a quiet closed room specially designed for the study. Each cage had a litter box, a fleece blanket, food, water, and a cardboard box for refuge. A diffuser with feline pheromones (Feliway classic; CEVA; Portugal) was placed in the room. All animals were denied food but not drink eight hours before anesthesia.

### 2.2. Anaesthetic Management

Before the procedure and using an online tool (www.randomization.com; accessed on 12 December 2020), cats were randomly allocated to one of two groups stipulating analgesic drugs to be administered: methadone 0.2 mg kg^−1^ IM (Semfortan; Dechra; Barcelona, Spain) in group control (CTR); methadone 0.1 mg kg^−1^ IM + SCC-E (lidocaine 0.3 mL kg^−1^ (Lidocaína 2%; Bbraun; Barcarena, Portugal) + preservative free morphine 0.1 mg kg^−1^ (Morfina 1%; Bbraun; Barcarena, Portugal) in sacrococcygeal epidural group (SCC-E).

In both groups, methadone was combined with dexmedetomidine 0.01 mg kg^−1^ (Sedadex; Dechra; Barcelona, Spain) and alfaxalone 1.5 mg kg^−1^ (Alfaxan; Dechra; Barcelona, Spain) in the same syringe and administered in a single injection into the epaxial lumbar musculature.

After adequate sedation and whilst pre-oxygenating with a face mask, an aseptic procedure was used to catheterize a cephalic vein, and the administration of a crystalloid fluid (Lactate-RingerVet; Bbraun) was initiated at a rate of 3 mL kg^−1^ h^−1^ and maintained throughout the procedure.

If within 10 min after IM premedication administration the depth of anaesthesia was inadequate for endotracheal intubation, an IV bolus of 1 mg kg^−1^ of alfaxalone was administered over 60 s and repeated as necessary until conditions were adequate. Thirty seconds before intubation, the larynx was desensitized with 0.1 mL of 2% lidocaine. After orotracheal intubation, to prevent leaks around the endotracheal tube during positive pressure ventilation, using a pediatric Mapleson D breathing system (subsequently used throughout the procedure), the endotracheal tube cuff was inflated.

Anaesthesia was maintained with sevoflurane (SevoFlo; Ecuphar; Sintra, Portugal)in 100% oxygen, delivered at the minimum flow required to maintain an inspired carbon dioxide fraction equal to zero during spontaneous ventilation. A sevoflurane vaporizer was adjusted to maintain an appropriate surgical anaesthetic depth throughout the procedure (eyes in an eccentric position, lack of palpebral reflex, mild jaw tone).

The eyes were lubricated after the onset of general anaesthesia, and then robenacoxib 2 mg kg^−1^ (Onsior, Elanco; Lisboa, Portugal) was given SC.

### 2.3. Blind Study

Four researchers were involved. Throughout the whole study period, only one investigator (AD) was aware of the treatment group each patient was allocated to. This researcher was responsible for drug preparation and administration, instrumentation, and performing the epidural technique in the SCC-E group. The other staff members involved in the intra- and post-operative assessments were not present in the induction room; they were only allowed to interact with the patients once they had been submitted to either the epidural administration or a sham process (clipping of the sacrococcygeal area and application of a fenestrated drape), in groups SCC-E and CTR, respectively. The same experienced surgeon, who was also blinded to study group allocation, performed the surgery on all animals.

### 2.4. Anaesthetic Drugs and Technique Used to Perform the Sacrococcygeal Epidural Anaesthesia

All cats involved in the study were placed in sternal recumbency, and the sacrococcygeal region was clipped and aseptically prepared. After verifying the presence of anal tone, the pelvic limbs were pulled forward and the interarcual space between the last sacral and the first coccygeal vertebrae was identified by palpation as described by O’Hearn and Wright [32].

The cats in group SCC-E were subsequently administered a sacrococcygeal epidural with 0.3 mL kg^−1^ of preservative-free lidocaine 2% (Lidocaina 2% B. Braun) and 0.1 mg kg^−1^ of preservative-free morphine 1% (Morfina 1% B. Braun) combined with NaCl 0.9% to a total volume of 1.5 mL in a 2 mL syringe, according to the following description.

As described by Otero et al., [34], the nerve stimulator (NS) (Stimuplex HNS12; Bbraun) was programmed to a fixed electrical current of 0.7 mA, frequency of 2 Hz, and pulse width of 0.1 ms. Then, a short bevel (30°) 22 G and 50 mm long unipolar insulated needle (Stimuplex A; BBraun) was connected to the NS and introduced in the SCC space. After the detection of muscle twitching in the horizontal plane of some segments of the tail, without involving the tail base or the perineal region, needle advancement was stopped, and the current was lowered to 0.3 mA to confirm that tail movement still occurred. Before needle withdrawal, the NS was turned off, and provided that no blood was aspirated nor resistance detected (using 0.5 mL of air bubble in the syringe), the anaesthetic solution was injected over 40 s.

With the animal still in sternal position for 10 min with hindlimbs abducted, anus relaxation and loss of anal reflex were verified using a thermometer in accordance with Credie and Luna [3]. Animals that did not experience loss of anal tone were excluded from further analysis, as it was considered that the epidural administration had failed. Animals in group CTR were submitted to the same waiting times (including the 5 min average time to perform epidural in the SCC-E group), under the same anaesthetic conditions.

### 2.5. Intraoperative Analgesia Assessments and Rescue Analgesia

Six surgical periods were defined for both groups: T_BASELINE_, after surgical field preparation, and before skin incision; T_OPEN_, cutaneous incision, dissection of subcutaneous tissues, and linea alba incision; T_OV1_, from traction to removal of the left ovarian pedicle; T_OV2_, from traction to the removal of the right ovarian pedicle; T_UTERINE_, traction and ligation of the uterus; T_CLOSE_, during celiorraphy and until the end of surgery.

Continuous electrocardiography (ECG), heart rate (HR), pulse oximetry, respiratory rate (RR), end-tidal carbon dioxide (ETCO2), and oesophageal temperature (°C) were monitored throughout the whole duration of anaesthesia using a pre-calibrated multiparametric monitor (Surgivet; BBraun). Non-invasive systolic (SAP), mean (MAP), and diastolic (DAP) arterial blood pressures were also monitored throughout anaesthesia, using a pre-calibrated high-definition oscillometric monitor (Memo Diagnostic; S + B medVet GmbH). Data were collected at 3 min regular intervals and then averaged to provide a single set of values for each anaesthetic event.

Additionally, the period between premedication to induction of general anaesthesia, the duration of anaesthesia (period of SEVO administration), and the duration of surgery (from the beginning of the cutaneous incision to the last suture) were also recorded.

When intra-surgical HR and/or SAP values increased 20% over T_BASELINE_ for 10 s, fentanyl (2.0 µg kg^−1^) (Fentadon; Dechra) was administered IV over 30 s. Whenever the variables did not return to the pre-stimulation values or increased again after 3 min, fentanyl IV bolus was repeated. The average consumption of fentanyl over the intraoperative period was recorded for each group.

If hypotension (defined as MAP < 60 mmHg) occurred, anaesthetic depth was decreased if possible, and a crystalloid solution (Lactate-RingerVet; BBraun) bolus at 5 mL kg^−1^ was administered over 5 min.

If hypotension persisted after the administration of two boluses of crystalloid fluid, a CRI of dopamine was administrated at 5 µg/kg/min and the animal was excluded from further analysis.

At the end of the surgery, sevoflurane was discontinued and extubation was performed after the swallowing reflex returned. Time to extubation (from turning off the vaporizer to extubation) and time to sternal recumbency (from extubation to sternal recumbency) were also registered. An anaesthetic recovery score was registered between extubation and return to sternal decubitus, according to a simple descriptive scale scored from 1 to 4 (poor, moderate, good, and excellent, respectively).

Cats were then allowed to recover in a warm and quiet environment. Once fully conscious, each cat was placed in its kennel room. Fluid therapy and heating support were discontinued when the rectal temperature was above 37.5 °C.

Each cat was discharged the day after surgery with a prescription of robenacoxib 2 mg kg^−1^ q 24 h (Onsior, Elanco; Lisboa, Portugal)) for the following 6 days.

### 2.6. Postoperative Pain Assessments and Rescue Analgesia

With the assistance of an experienced veterinary nurse, also blinded to the treatment groups, the assessments were made before premedication (T_BEFORE_) and 1, 2, 3, 4, 6, and 8 h (T_1_, T_2_, T_3_, T_4_, T_6_, and T_8_, respectively) after extubation and two methods of pain assessment were used:

1. UNESP-Botucatu multidimensional composite pain scale (UNESP-Botucatu-MCPS) with increasing pain levels reflected by a score from 0 to 27, based on specific behaviours, posture, attitude, and reactions to physical contact. Due to the difficulty in measuring blood pressure in non-anaesthetized and/or sedated cats [37], this parameter was not evaluated postoperatively (sub-scale 3-physiological variables).
2. Wound sensitivity by measuring the minimal mechanical nociceptive threshold (MNT) using a pneumatic device (ELECALL ELK-50 Digital Dynamometer Force Measuring), with an 8 mm round, flat tip, applied near the surgical wound.

Each cat was first observed in the cage without being disturbed and evaluations were done in the following order: RR (breaths minute^−1^) by observation of chest wall movement; after opening the cage, HR (beats minute^−1^) by chest auscultation using a stethoscope. It was then gently handled, offered a small amount of food, and its behaviour and posture assessed to assign the UNESP-Botucatu-MCPS score.

Finally, the cats were removed from the cage, and MNT was measured by placing the tip 1 cm away from the surgical wound, at a cranial and caudal location on each side of the suture and applying constant pressure, perpendicular to the surface of the skin. With an interval of 30 s between stimuli, an average N value of the 4 measurements for each animal was obtained and a maximum limit value of 20 N (10 N = 1 kgf) was established to be registered.

When a painful response was noticed, considered as a sharp head turn, aggressive behavior, or vocalization, the tip was removed right away, holding the peak reading, and the response was recorded as MNT (registered in Newtons) (N).

When the UNESP-Botucatu-MCPS score was ≥6/27 in each pain assessment, an IV bolus of 0.2 mg kg^−1^ of methadone was administered.

### 2.7. Statistical Analysis

The distribution of the variables was assessed based on the Shapiro–Wilk test. Comparison between groups was performed using independent samples *t*-test if the assumption of normality was verified, or the Mann–Whitney test. The chi-square test of independence and exact Fisher’s test were used to compare groups according to recovery quality. The repeated measures analysis of variance (ANOVA) was used to compare groups over time. In all analyses, a significance level of 0.05 was considered and the program JMP 14.0, SAS Institute, North Carolina, USA, 2018 was used.

Based on previous case studies, considering 4 individuals in each group and considering the total amount of fentanyl administered as an outcome, G*Power software (Version 3.1.9.3; Axel Buchner; Edgar Erdfelder; Franz Faul; Albert-Georg Lang; Düsseldorf, Germany) calculated the sample size. The power calculation was performed using a two-tailed *t*-test with a power of 0.95, alpha error of 0.05, and effect size of 3, calculated based on the mean and standard deviation of the two preliminary groups. The results of the analysis indicated that a sample size of 14 individuals (seven in each group) would be sufficient to detect differences between the two groups. However, we assigned 10 cats to each group to account for potential losses because of individual case management.

## 3. Results

Out of the 29 cats that were recruited for our study, nine animals were excluded due to aggressive behaviour (*n* = 8) and pregnancy (*n* = 1). The remaining 20 cats were divided into 10 animals per group. Demographic data, anaesthesia/surgery duration, and times to extubation and sternal recumbency did not differ significantly among groups (Table 1).

Considering the mean weight of the population of our study, the concentration of lidocaine was approximately 1.2% (12 mg/mL). All animals in the SCC-E group were found to have tail muscle contractions during the nerve stimulator test and a lack of anal tone after epidural administration. No animals in this group showed nociceptive signs during epidural administration and, at the end of the surgery, none showed signs of hind limb motor block or incidence of hind limb ataxia superior to the CTR group.

No animals were excluded after study group allocation or developed hypotension or other cardiorespiratory and/or neurological perioperative complications.

**Figure 1:**
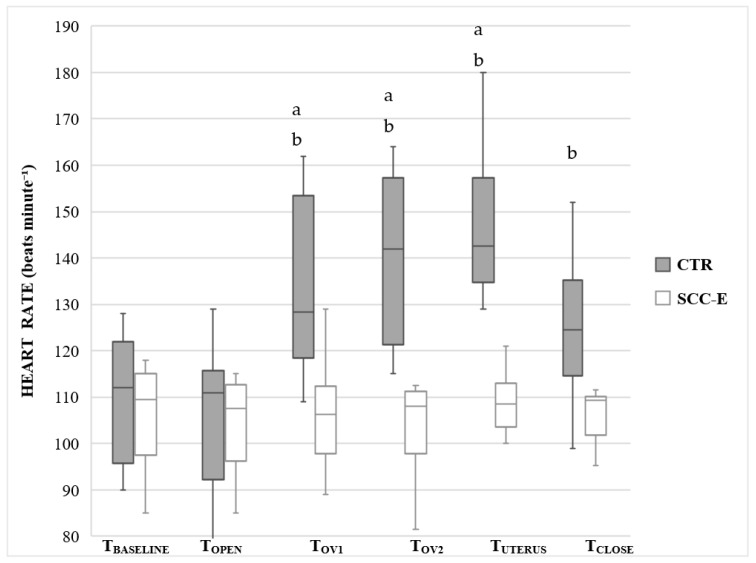
Heart rate (HR) in cats undergoing ovariohysterectomy with (SCC-E group) or without (CTR group) sacrococcygeal epidural (*n* = 10 in each group). Times: T_BASELINE_, prior to skin incision; T_OPEN_, abdominal skin incision; T_OV1_, from traction to removal of left ovary; T_OV2_, from traction to removal of right ovary; T_UTERUS_, traction and ligation of the uterus; T_CLOSE_, during celiorraphy until the end of surgery. The horizontal line inside the boxes denotes the median value, while the upper and lower whisker bounds denote the maximum and minimum values. The boxes show the interquartile ranges. ^a^ Significant difference compared with T_BASELINE_ (*p*-values were <0.0001, <0.0001, and <0.0001 respectively). ^b^ Significant difference compared with SCC-E group (*p*-values were =0.0001, =0.0001, <0.0001, and =0.014 to T_OV1_, T_OV2_, T_UTERUS,_ and T_CLOSE_, respectively).

**Figure 2:**
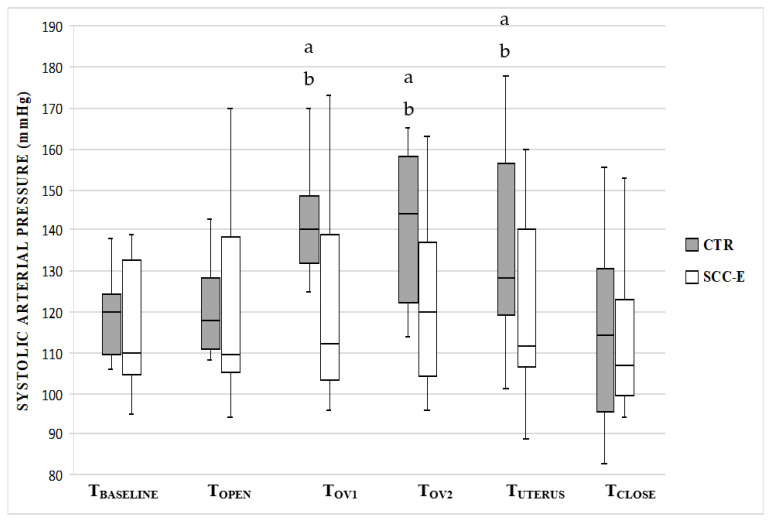
Systolic arterial pressure (SAP) in cats undergoing ovariohysterectomy with (SCC-E group) or without (CTR group) sacrococcygeal epidural (*n* = 10 in each group). Times: T_BASELINE_, prior to skin incision; T_OPEN_, abdominal skin incision; T_OV1_, from traction to removal of left ovary; T_OV2_, from traction to removal of right ovary; T_UTERUS_, traction and ligation of the uterus; T_CLOSE_, during celiorraphy until the end of surgery. The horizontal line inside the boxes denotes the median value, while the upper and lower whisker bounds denote the maximum and minimum values. The boxes show the interquartile ranges. ^a^ Significant difference compared with T_BASELINE_ (*p*-values were 0.009 and 0.006 for T_OV1_ and T_OV2_, respectively). ^b^ Significant difference compared with the SCC-E group (*p*-values were 0.040 and 0.037 for T_OV1_ and T_OV2_, respectively).

**Figure 3:**
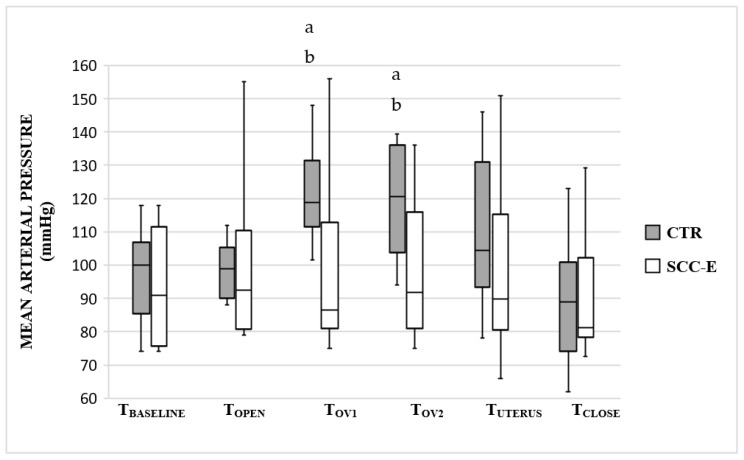
Mean arterial pressure (MAP) in cats undergoing ovariohysterectomy with (SCC-E group) or without (CTR group) sacrococcygeal epidural (*n* = 10 in each group). Times: T_BASELINE_, prior to skin incision; T_OPEN_, abdominal skin incision; T_OV1_, from traction to removal of the left ovary; T_OV2_, from traction to removal of the right ovary; T_UTERUS_, traction and ligation of the uterus; T_CLOSE_, during celiorraphy until the end of surgery. The horizontal line inside the boxes denotes the median value, while the upper and lower whisker bounds denote the maximum and minimum values. The boxes show the interquartile ranges. ^a^ Significant difference compared with T_BASELINE_ (*p*-values were 0.018 and 0.012 for T_OV1_ and T_OV2_, respectively). ^b^ Significant difference compared with group SCC-E (*p*-values were 0.021 and 0.019 for T_OV1_ and T_OV2_, respectively).

There were no significant changes in HR, SAP, and MAP between all intra-surgical assessment times in group SCC-E whereas Group CTR had a significant increase from baseline in overall mean HR (*p* < 0.0001), SAP (*p* = 0.0031), and MAP (*p* = 0.0014) (Figure 1, Figure 2 and Figure 3).

Comparing groups for each surgical period, HR was significantly higher in the group CTR in T_OV1_, T_OV2_, T_UTERUS,_ and T_CLOSE_ compared with group SSC-E (Figure 1). The same occurred for SAP and MAP during T_OV1_ and T_OV2_ (Figure 2 and Figure 3).

The mean (±SD) value of fentanyl required in group CTR (18.7 ± 3.7 μg) was significantly higher compared with group SCC-E (4.9 ± 2.5 μg) (*p* < 0.001) (Table 2).

There were no significant differences in temperature values [mean (range)] between groups [37.6 °C (36–37.6 °C) and 37.8 °C (36.3–37.9 °C) in groups CTR and SCC-E, respectively] (*p* > 0.05) (Table 3).

No significant respiratory (ETCO2 and RR) differences between both groups were observed (Table 3).

Recovery quality score tended to be higher in group SCC-E, as 8/10 animals were classified with excellent recovery quality, compared to 4/10 animals in group CTR, although without significant differences between groups (*p* = 0.139) (Table 4).

**Figure 4:**
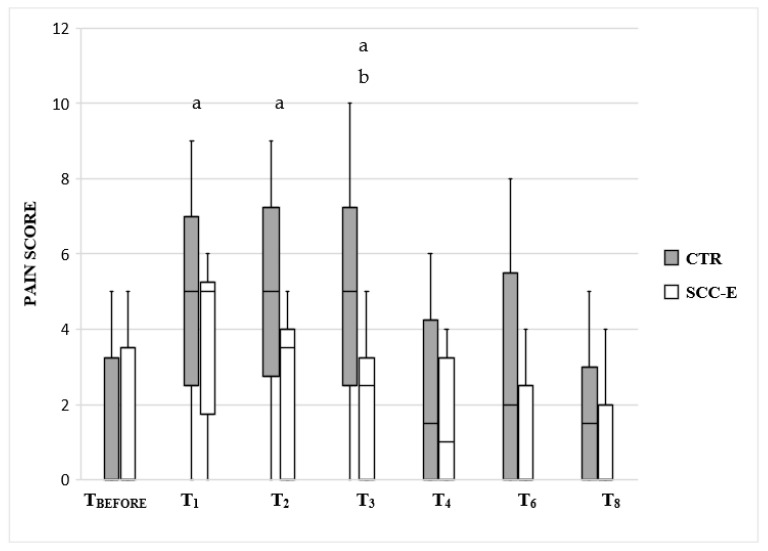
UNESP-Botucatu-MCPS pain scale recorded for 8 h after surgery in cats undergoing ovariohysterectomy with (SCC-E group) or without (CTR group) sacrococcygeal epidural (*n* = 10 in each group). Times: T_BEFORE_, prior to premedication, T_1_, T_2_, T_3_, T_4_, T_6_, and T_8_ = 1 h, 2 h, 3 h, 4 h, 6 h, and 8 h after surgery completion, respectively. The horizontal line inside the boxes denotes the median value, while the upper and lower whisker bounds denote the maximum and minimum values. The boxes show the interquartile ranges. ^a^ Significant differences compared with T_BEFORE_ (*p* = 0.0045, 0.0004, and 0.0034 for T_1_, T_2_, and T_3_, respectively). ^b^ Significantly superior compared to the SCC-E group (*p* = 0.027).

There were no significant changes in UNESP-Botucatu-MCPS pain scores at all assessments in group SCC-E. Compared to T_BEFORE_, the CTR group had significantly higher pain scores at T_1_, T_2_, and T_3,_ and compared to group SCC-E, pain scores were higher for the CTR group at all times with a significant difference in T_3_ (*p* = 0.027) (Figure 4).

A higher methadone consumption was observed in group CTR when compared to SCC-E, although without significant differences (*p* = 0.1904) (Table 5). All animals receiving postoperative analgesic rescue with methadone decreased their pain scores in the next evaluation period.

**Figure 5:**
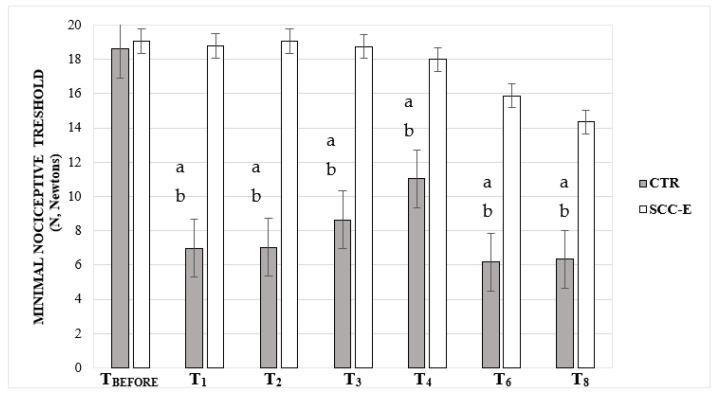
Median and standard deviation of perianesthetic values of mechanical nociceptive thresholds (MNT) in cats undergoing ovariohysterectomy with (SCC-E group) or without (CTR group) sacrococcygeal epidural (*n* = 10 in each group). Times: T_BEFORE_, prior to premedication, T_1_, T_2_, T_3_, T_4_, T_6_, and T_8_ = 1 h, 2 h, 3 h, 4 h, 6 h, and 8 h after the end of the surgery, respectively. The horizontal line inside the boxes denotes the median value, while the upper and lower whisker bounds denote the maximum and minimum values. The boxes show the interquartile ranges. ^a^ Significant differences compared with T_BEFORE_ (*p* = 0.0002, 0.0008, 0.0018, 0.0161, 0.0007, 0.0011 for T_1_, T_2_, T_3_, T_4_, T_6_, and T_8_, respectively). ^b^ Significant differences compared with the SCC-E group (*p* = 0.0001, 0.0009, 0.0025, 0.0284, 0.0121, 0.0412 for T_1_, T_2_, T_3_, T_4_, T_6_, and T_8_, respectively).

Compared to T_BEFORE_, the CTR group had significantly lower MNT values at all assessment points except for T_8_ while in contrast, there were no significant changes in MNT values at all assessments in group SCC-E. Moreover, the overall MNT in the SCC-E was significantly higher at all assessment points compared to the CTR group (Figure 5).

## 4. Discussion

The primary finding of this study is that compared to cats that received systemic analgesia alone, cats who received SCC-E administration of lidocaine and morphine showed evidence of greater regulation of nociceptive response to surgical stimulation and required less perioperative rescue analgesia. The study used young, healthy female cats to imitate the normal clinical scenario where elective sterilization operations are frequently carried out. Each group of cats included cats that were around the same age and weight, which reduced the possibility of confounding variables. The differences in nociceptive response evidenced by the different haemodynamic responses between the two groups in our study suggest that epidural analgesia had an important influence on the control of nociceptive stimulus related to surgical trauma. This likely happened due to the capacity of drugs injected in the epidural space to prevent the activation and traffic of afferent neuronal impulses caused by the nociceptive surgical stimulus to the CNS, whereas systemic administration of opioids does not prevent the transmission of impulses to the CNS and only just modulates the synaptic neurotransmission of the nociceptive stimulus as soon as it reaches the CNS.

Our findings are in line with those made earlier by other authors, who discovered that epidural lidocaine allowed OHE in cats with adequate analgesia and within an acceptable range for cardiovascular and respiratory changes [21,32,38].

The significant cardiovascular changes in group CRT occurred during specific surgical periods known to be responsible for the most significant nociceptive stimulus during an OHE [7,39]. In contrast, the unchanged intraoperative overall HR and SAP values observed in group SCC-E for all surgical times suggest that the epidural technique used in this study allowed the inhibition of the haemodynamic response.

Few studies report on the CNS origin of the innervation of small animal ovaries. Rosengren and Sjöuberg [40] showed that in cats, postganglionic sympathetic innervation reaches the oviduct, uterus, and vagina via the hypogastric nerve and adrenergic neurons originating from the uterovaginal junction. However, they did not determine the contribution of the spinal segments responsible for ovarian innervation. In 1991, Chien et al. [41] concluded that the canine ovary receives sensory nerve fibres originating and widely spread between the T10 and L4 medullary segments and found higher concentrations near the thoracolumbar junction, from T13 to L3; consequently, to block all relevant nerve fibres, the local anaesthetic solution should be distributed at least between the former segments. Although there are no similar studies in the feline species, the fact that the embryonic origin of the canine ovaries is similar to cats, the authors of the present study hypothesize that the same applies to this species [42,43].

Four animals in the SCC-E group required intra-operative analgesic rescue due to haemodynamic response. One possible explanation may be related to the technique used, since assuming a 5% probability of error, the positive predictive value for successful epidural injection may extend from 78.8% to 99.8% [35]. Another explanation, more plausible from the authors’ point of view, is related to the possible variable distribution between individuals and insufficient cranial distribution of the anaesthetic solution to block the medullary segments from T10 to L4 in the above-mentioned animals. We speculate that in these animals the cranial distribution of the anaesthetic solution was insufficient to reach the medullary segments that receive all the afferent nerve fibres of the ovaries (T10 to L4) [41]. However, the reduced number of rescue analgesia events in these animals compared to the CTR group suggests that it was probably sufficient to block the medullary segments that receive the majority of these fibres (T13-L3) [41]. Another possible explanation is the volume of physiological saline added to the lidocaine/morphine mixture that led to a lidocaine dilution effect varying between animals according to their weight, which may have allowed different local anaesthetic onset and duration of effect times [38,44].

Three animals belonging to the SCC-E group received post-surgical analgesic rescue, this happened possibly due to several hypotheses already described above since these animals also required intraoperative analgesia. Considering that two of the animals in the SCC-E group required analgesic rescue within the first hour of assessment, another hypothesis is that, despite using the UNESP-Botucatu-MCPS, the research misinterpreted signs of dysphoria as being of pain.

Although not statistically different, time to extubation and time to sternal recumbency was prolonged in group SCC-E. This could be related to a sedating effect of the epidural drugs due to systemic absorption, but another possible explanation is that slower anaesthetic recovery after epidural analgesia is related to a more pronounced postoperative control of pain, as described in humans [45].

To access postoperative pain after OHE in cats, the UNESP-Botucatu MCPS is a reliable and validated tool [46]. The implementation of minimal mechanical nociceptive threshold (MNT) has also been described in some pain assessment studies of various analgesic protocols performed in cats [9,10,47,48] but it is important to note that the pain scale and MNT measure two different aspects of nociception: the first essentially measures spontaneous pain and the second measures pain induced by a pressure device. This may explain the significant changes in MNT values in the CTR group at all time points compared to the T_BEFORE_ value and is consistent with what also happened in the study by Slingsby et al. [9]; by studying the effectiveness of methadone use in combination with medetomidine in OHE in cats, the authors found the development of secondary mechanical hyperalgesia through increased pressure sensitivity around the suture, despite the use of methadone on the premedication. The same happened in other systemic analgesia protocols [12,47,48]. The absence of significant changes in MNT values in the SCC-E group between all evaluation periods contrasted with those observed in these studies and demonstrates that the epidural technique used in this study was effective in controlling mechanical hyperalgesia. The basal MNT values observed in the present study (16.9 ± 3.5 and 18.6 ± 1.8 for CTR and SCC-E group, respectively) were different from those obtained at this same time in other studies that used this method to evaluate postoperative pain. Slingsby et al. [9] obtained mean values of 3.3 ± 0.8 N, Benito et al. [48] recorded average values of 13–15 N, and Benito et al. [47] between 11 and 13 N. There could be various possible explanations for these differences. On one hand, different devices were used for these measurements and these differences may be justified by the difference in the devices’ sensitivity. On the other hand, the different methodologies used in these studies, namely regarding the number of measurements, probe application speed, measurement location, and time interval between measurements, may be the most plausible reason for these differences. Moreover, the evaluation of the response is not free of any subjectivity as it is inherent in the operator’s perception. In our study, an 8 mm diameter round-shaped flat tip device was used. Since Pressure = Force (N)/Area (mm^2^), using a force of 20 N (2 kgf), the 8 mm tip used (50.24 mm^2^) exerted a pressure of 4 kgf/cm^2^. The existence of measuring tips with different shapes and diameters between studies is another important factor that could lead to these differences.

An important factor that influences the cranial distribution of anaesthetic solution in the epidural space is volume [49,50]. A recent cadaver study used 12 dogs to compare the cranial distribution of local anaesthetic through epidural administration using the LS and SCC approaches, leading to the conclusion that there were no significant differences in cranial contrast fluid distribution between the two approaches [51]. Otero and Portela [35] reported a volume of lidocaine between 1 and 1.5 mL per cat, using the lumbosacral approach to reach an extension between L1-T9, although, according to the authors’ knowledge, there is no published literature regarding the cranial distribution of local anaesthetic when using SCC-E in cats and that knowledge would be important in the future as a guideline for the administration of anaesthetic drugs by this route.

The choice of morphine dosage in this study was based on the commonly used clinical dose and has shown to be effective in treating perioperative pain in dogs and cats [21,52,53]. Although epidural morphine administration can cause side effects such as pruritus and urinary retention [54,55], the benefits of its use outweigh these uncommon side effects. Castro et al. [56] demonstrated that analgesia produced by the lumbosacral epidural administration of morphine (0.1 mg kg ^−1^) lasts over 12 h. Pypendop et al. [57] compared the thermal nociceptive analgesia of epidural morphine (0.1 mg kg ^−1^) and buprenorphine (12 mcg kg ^−1^) in cats and reported that thermal thresholds remained higher and lasted over 16 h, compared to buprenorphine (10 h).

The doses and volume of lidocaine used in this study were chosen based on previous cases where the epidural administration produced clinically effective anti-nociceptive effect in adult cats submitted to OHE (weighing 3–4 kg and with an ideal body condition of 4–5/9). This dose (6 mg kg^−1^) is less than that considered to be the safe threshold to avoid neurological (11.7 mg kg^−1^) and cardiovascular (47.3 mg kg^−1^) toxicity in cats [58,59] and is regarded as a safe dose [60].

There are some important limitations to recognize in this study: it would be ideal to add a group receiving epidural administration without systemic analgesia during premedication to better reflect the effects of the former, however, this is against ethical committee requirements. Another limitation is the use of the saline solution to achieve a fixed total volume to be administered epidurally. This may have led to different lidocaine dilutions from one animal to the other, in accordance with their weight; this variation was likely minimal given the weight distribution of our population, but it could have conceivably caused differing local anaesthetic onset and duration of effect. Considering the mean weight of the population of our study, the concentration of lidocaine was approximately 1.2% (12 mg/mL). Despite the significant differences observed between groups, the real magnitude of the nociceptive response may be attenuated after the first opioid administrations in those animals that received analgesic rescue in the intra and postoperative periods. The MNT measurements were performed using a pneumatic device which has not been yet studied under clinical conditions.

## 5. Conclusions

Based on our results, the SCC-E administration of lidocaine 2% with morphine 1%, diluted with NaCl 0.9% to a total volume of 1.5 mL, is a reasonable option to provide perioperative analgesia in cats submitted to OHE, compared to a systemic protocol alone. This study indicates that the implementation of SCC-E could be a simple and effective technique to control perioperative pain in cats submitted to OHE. Further studies will be needed to evaluate local anaesthetic distribution through a SCC approach in cats.

## Figures and Tables

**Table 1:** Demographic data, duration of anaesthesia time, duration of surgery, times until extubation and until sternal recumbency in cats undergoing ovariohysterectomy with (SCC-E group) or without (CTR group) sacrococcygeal epidural (*n* = 10 in each group).

VARIABLE	CTR	SCC-E	*p*-Value
Age (months, median (IQR))	11.5 (6.25)	12.0 (20.0)	0.393
Weight (kg, mean ± SD)	3.0 ± 0.5	3.1 ± 0.5	0.621
Body condition (1–9, median (IQR))	6.0 (2.0)	6.0 (1.0)	0.353
Duration of the anaesthesia (minutes, median (IQR))	40.5 (6.5)	44.5 (4.8)	0.063
Duration of the surgery (minutes, median (IQR))	14.5 (3.8)	16.0 (4.3)	0.315
Time to extubation (minutes, median (IQR))	7.5 (8.5)	8.5 (24.0)	0.393
Time to sternal recumbency (minutes, median (IQR)	2.0 (11.0)	11.5 (34.8)	0.143

SD, standard deviation; IQR—interquartile range.

**Table 2:** Total number of animals that required rescue analgesia at each surgical event and mean ± SD amount of fentanyl administered in cats undergoing ovariohysterectomy with (SCC-E group) or without (CTR group) sacrococcygeal epidural (*n* = 10 in each group).

Number of Analgesic Rescues						Animals with Rescues	Total Amount of Methadone (μg): Mean ± SD
	T_OPEN_	T_OV1_	T_OV2_	T_UTERUS_	T_CLOSE_		
**CTR**	0	8	8	8	5	10/10 (100%)	18.7 ± 3.7 ^a^
**SCC-E**	1	0	2	3	1	4/10 (40%)	4.9 ± 2.5

Times: T_OPEN_, abdominal skin incision; T_OV1_, from traction to removal of left ovary; T_OV2_, from traction to removal of right ovary; T_UTERUS_, traction and ligation of the uterus; T_CLOSE_, during celiorraphy until the end of surgery. ^a^ Significant difference compared with the SCC-E group (*p* = 0.001).

**Table 3:** Mean and standard deviation (SD) of intra-surgical respiratory rate (RR), end-tidal carbon dioxide (ETCO2), expired fraction sevoflurane (SEVO), and temperature (TEMP) values in cats undergoing ovariohysterectomy with (SCC-E group) or without (CTR group) sacrococcygeal epidural (*n* = 10 in each group).

Variable	Group	T_BASELINE_	T_OPEN_	T_OV1_	T_OV2_	T_UTERUS_	T_CLOSE_
**RR** (breaths minute^−1^ ± SD)	SCC-E	23 ± 6	22 ± 6	22 ± 6	21 ± 5	19 ± 7	20 ± 8
	CTR	29 ± 6	28 ± 6	26 ± 6	23 ± 5	23 ± 7	23 ± 8
**ETCO_2_** (mmHg ± SD)	SCC-E	41 ± 5	40 ± 5	41 ± 5	39 ± 5	40 ± 7	40 ± 6
	CTR	39 ± 4	36 ± 6	36 ± 5	36 ± 6	34 ± 9	36 ± 5
**TEMP** (C° ± SD)	SCC-E	36.8 ± 0.6	36.9 ± 0.6	36.8 ± 0.6	36.7 ± 0.6	36.6 ± 0.6	36.5 ± 0.6
	CTR	36.7 ± 0.3	36.8 ± 0.4	36.6 ± 0.5	36.4 ± 0.4	36.3 ± 0.3	35.9 ± 1.2
**SEVO** (% ± SD)	SCC-E	2 ± 0	2 ± 0.5	2 ± 0	2 ± 0	2 ± 0.5	1.5 ± 0.5
	CTR	2 ± 0	2 ± 0.5	2 ± 1	2 ± 0.5	2 ± 0	2 ± 0.5

T_BASELINE_, prior to skin incision; T_OPEN_, abdominal skin incision; T_OV1_, from traction to removal of left ovary; T_OV2_, from traction to removal of right ovary; T_UTERUS_, traction and ligation of the uterus; T_CLOSE_, during celiorraphy until the end of surgery.

**Table 4:** Simple descriptive scale (SDS) scores were used to assess recovery quality score recovering from anaesthesia (between extubating until return to sternal decubitus) in cats undergoing ovariohysterectomy, with (group SCC-E) or without (group CTR) sacrococcygeal epidural (*n* = 10 in each group).

Score	Recovery Quality	CTR	%	SCC-E	%
**1**	Poor: Evident signs of excitement during recovery to sternal recumbency such as sudden movements around and lack of awareness of the surrounding environment; growl that does not respond to the animal’s touch.	0/10	0	0/10	0
**2**	Moderate: slight signs of excitement during recovery to sternal recumbency such as sudden movements around and lack of awareness of the surrounding environment; growling that does respond to the animal’s touch.	1/10	10	1/10	10
**3**	Good: mild signs of excitement that resolve quickly and the animal becomes calm during recovery to sternal recumbency.	5/10	50	1/10	10
**4**	Excellent: calm and relaxed animal during recovery to sternal recumbency.	4/10	40	8/10	80

**Table 5:** Number of analgesic rescues at each postoperative time, receiving methadone IV bolus, whenever the UNESP-Botucatu-MCPS pain scale was ≥ 6 in cats undergoing ovariohysterectomy with (SCC-E group) or without (CTR group) sacrococcygeal epidural (CTR group) (*n* = 10 in each group).

	T_1_	T_2_	T_3_	T_4_	T_6_	T_8_	Total Number of Rescue Animals	Total Amount of Methadone (mg)
**CTR**	5	3	3	1	2	0	7/10 (70%)	7.4
**SCC-E**	2	0	0	1	0	0	3/10 (30%)	1.88

## Data Availability

Not applicable.
